# Conjugation to a SMAC mimetic potentiates sigma-2 ligand induced tumor cell death in ovarian cancer

**DOI:** 10.1186/1476-4598-13-50

**Published:** 2014-03-07

**Authors:** Gunjal Garg, Suwanna Vangveravong, Chenbo Zeng, Lynne Collins, Mary Hornick, Yassar Hashim, David Piwnica-Worms, Matthew A Powell, David G Mutch, Robert H Mach, William G Hawkins, Dirk Spitzer

**Affiliations:** 1 Department of Obstetrics and Gynecology, Division of Gynecologic Oncology, Washington University School of Medicine, St. Louis, MO 63110, USA; 2 Department of Radiology, Division of Radiological Sciences, Washington University School of Medicine, St. Louis, MO 63110, USA; 3 Department of Radiology, University of Pennsylvania, Chemistry Building, Room 283, 231 S. 34th St, Philadelphia, PA 19104, USA; 4 Departments of Cell Biology & Physiology, Developmental Biology, Molecular Imaging Center, Mallinckrodt Institute of Radiology, BRIGHT Institute, St. Louis, MO 63110, USA; 5 Department of Surgery, Washington University School of Medicine, 660 S. Euclid Avenue, Campus Box 8109, St. Louis, MO 63110, USA; 6 The University of Texas M.D. Anderson Cancer Center, Cancer Systems Imaging Department, Division of Diagnostic Imaging, T. Boone Pickens Academic Tower, 1400 Pressler Street, Unit 1479, Houston, TX 77030, USA; 7 Siteman Cancer Center, Washington University School of Medicine, St. Louis, MO 63110, USA; 8 Britton Chance Professor of Radiology, Director of Radiochemistry, University of Pennsylvania, Chemistry Building, Room 283, 231 S. 34th St, Philadelphia, PA 19104, USA

**Keywords:** Sigma-2 ligand, SMAC-peptidomimetic, Small molecule, Drug conjugate, Targeted drug delivery, Apoptosis, Ovarian cancer

## Abstract

**Background:**

Drug resistance is a significant problem in the treatment of ovarian cancer and can be caused by multiple mechanisms. Inhibition of apoptosis by the inhibitor of apoptosis proteins (IAPs) represents one such mechanism, and can be overcome by a mitochondrial protein called second mitochondria-derived activator of caspases (SMAC). We have previously shown that the ligands of sigma-2 receptors effectively induce tumor cell death. Additionally, because sigma-2 receptors are preferentially expressed in tumor cells, their ligands provide an effective mechanism for selective anti-cancer therapy.

**Methods:**

In the current work, we have improved upon the previously described sigma-2 ligand SW43 by conjugating it to a pro-apoptotic small molecule SMAC mimetic SW IV-52, thus generating the novel cancer therapeutic SW IV-134. The new cancer drug was tested for receptor selectivity and tumor cell killing activity in vitro and in vivo.

**Results:**

We have shown that SW IV-134 retained adequate sigma-2 receptor binding affinity in the context of the conjugate and potently induced cell death in ovarian cancer cells. The cell death induced by SW IV-134 was significantly greater than that observed with either SW43 or SW IV-52 alone and in combination. Furthermore, the intraperitoneal administration of SW IV-134 significantly reduced tumor burden and improved overall survival in a mouse xenograft model of ovarian cancer without causing significant adverse effects to normal tissues. Mechanistically, SW IV-134 induced degradation of cIAP-1 and cIAP-2 leading to NF-қB activation and TNFα-dependent cell death.

**Conclusions:**

Our findings suggest that coupling sigma-2 ligands to SMAC peptidomimetics enhances their effectiveness while maintaining the cancer selectivity. This encouraging proof-of-principle preclinical study supports further development of tumor-targeted small peptide mimetics via ligands to the sigma-2 receptor for future clinical applications.

## Background

Ovarian cancer is the second most common gynecologic malignancy in the United States. However, it is the deadliest of all gynecologic cancers; of the 28,080 expected deaths from gynecologic malignancies annually, about 50% or 14,030 will be from ovarian cancer [1]. Most patients respond well to the initial treatment with surgical de-bulking and combination chemotherapy [2]. The disease eventually recurs in a large number of patients leading to death mainly due to the lack of effective treatments against drug resistant disease [3]. There is a clear need to identify novel therapeutic agents that target critical drug resistance pathways to improve survival in ovarian cancer.

Apoptosis or programmed cell death is a cell suicide mechanism which plays a critical role in the development and homeostasis in vertebrates and invertebrates [4]. Inhibition of apoptosis can prevent cancer cell death and promote the development of drug resistance in various malignancies [5]. The inhibitor of apoptosis proteins (IAPs) are among the principal molecules that contribute to this phenomenon [6]. The anti-apoptotic activity of IAPs can be overcome by second mitochondria-derived activator of caspases (SMAC), a mitochondrial protein which is released into the cytoplasm in response to apoptotic stimuli [7]. A number of compounds that mimic the function of the SMAC proteins have been described recently with striking pro-apoptotic activity reported in vitro and in vivo [8-11].

We have previously described the development and potential diagnostic and therapeutic application of sigma-2 ligands for various types of malignancies [12-15], and shown that sigma-2 receptor ligands bind to the PGRMC1 protein complex [16]. Sigma-2 ligands are especially suitable for purposes of diagnostic imaging and therapeutic targeting of solid tumors due to their high selectivity for tumor cells in vivo [17,18]. Furthermore, by virtue of their rapid internalization and binding to the sigma-2 receptor [19], these ligands represent excellent candidates for selective delivery of anticancer drugs into the tumor cells [14]. Taking advantage of these two unique properties of sigma-2 ligands, we have generated dual-domain therapeutics, wherein sigma-2 ligands additionally function as targeting domains for a cancer-selective delivery of effector molecules such as pro-apoptotic peptides into the tumor cells (12). One such compound is SW IV-134, which is a conjugate of the sigma-2 ligand SW43 and a small molecule SMAC mimetic SW IV-52. In our current report, we describe in detail the in vitro characterization of SW IV-134 and explore its effectiveness in preclinical models of ovarian cancer.

## Results

### The novel sigma-2/SMAC drug conjugate SW IV-134

**Figure 1:**
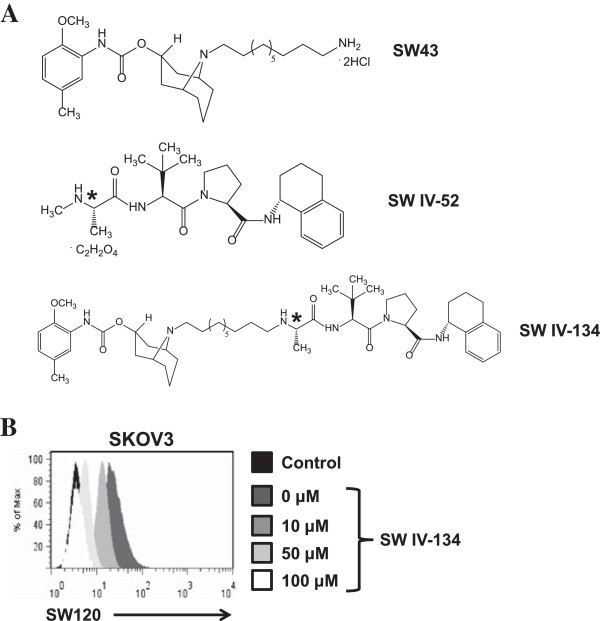
**Compound structures and cell binding characteristics of SW IV-134. (A)** The chemical structures of sigma-2 ligand SW43, the SMAC peptidomimetic SW IV-52, and the chemical conjugate SW IV-134. Note that the chirality of SW IV-52 is fully retained in the drug conjugate (asterisks). **(B)** SW IV-134 prevents uptake of the fluorescently labeled sigma-2 ligand SW120 in a dose-dependent fashion. SKOV3 cells were pretreated with increasing concentration of SW IV-134, followed by incubation with SW120, prior to analysis by flow cytometry.

In continuation of our work on the cancer-selective delivery of drug cargoes via the sigma-2 delivery platform, we extended our efforts from peptides to peptidomimetics. One of the key modulators of drug resistance in many types of human malignancies is intracellular XIAP. We built on the previously described small molecule XIAP-inhibitor, SMAC, derived from the endogenous inhibitor of the intrinsic apoptosis pathway [20]. The SMAC peptidomimetic SW IV-52 exactly matches the published structure of this small molecule XIAP-inhibitor [20], and was chemically linked to the sigma-2 receptor selective ligand SW43 (see M&M for details on drug synthesis), giving rise to the novel drug conjugate SW IV-134 (Figure 1A). Based on the parental compounds, this new cancer drug was predicted to selectively find its target (via its delivery moiety SW43) and enhance SW43 induced apoptosis according to the activity profile of its cargo (SW IV-52). The structural features of the delivery vehicle and the drug cargo were well preserved in the chemically combined cancer therapeutic SW IV-134 (Figure 1A).

In order to verify its unaltered target recognition properties, the binding affinity of SW IV-134 to the sigma-2 receptor was determined using previously established methods employing rat liver membrane homogenates (see M&M and Ref [19] for details on receptor binding determinations). SW IV-134 exhibited an excellent sigma-2 receptor binding profile (K_i_ sigma-2: 22.6 ± 1.8 nM), which was comparable to that of the parental molecule SW43 (K_i_ sigma-2: 7.1 ± 1.3 nM) (Additional file 1: Table S1). As another means to probe for the ability of SW IV-134 to bind to the sigma-2 receptor, we performed a competition assay using intact ovarian cancer cells and the fluorescently labeled SW43 homolog SW120 [21]. Pretreatment of the ovarian cancer cell line SKOV3 with SW IV-134 resulted in a dose-dependent reduction of SW120 signal intensity and was completely blocked with the highest concentration of SW IV-134 used in this assay system (Figure 1B). Of note, a similar effect was seen with SW43 alone, while SW IV-52 (the SMAC peptidomimetic) was nearly incapable of blocking the uptake of SW120 (Additional file 2: Figure S1). Together, these data support the notion that the receptor binding properties of the sigma-2 ligand SW43 were not altered following conjugation to the SMAC mimetic SW IV-52.

### SW IV-134 enhances cancer cell death

**Figure 2:**
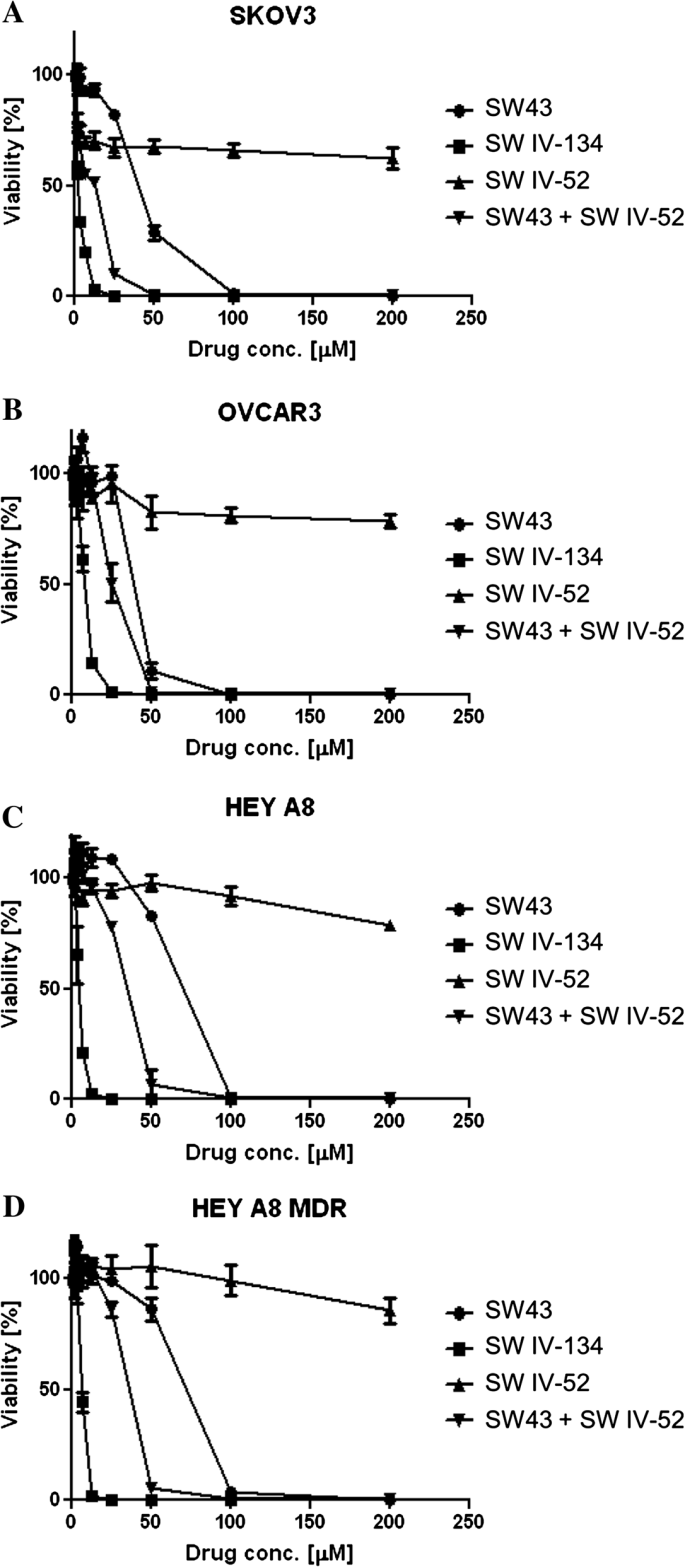
**Conjugation of the SMAC mimetic SW IV-52 to SW43 significantly enhances the killing potential of the individual components. (A)** SKOV3, **(B)** OVCAR3 **(C)** HEY A8 and **(D)** HEY A8 MDR cells were treated with increasing doses of SW43, SW IV-52, SW43 plus SW IV-52, and SW IV-134 for 18 hours. Cell viability was determined by CellTiter-Glo assays [p < 0.015 **(A)**, NS **(B)**, p < 0.017 **(C)**, p < 0.034 **(D)**, one-way ANOVA].

We tested the killing activity of SW43, SW IV-52, a combination of individual components of SW43 and SW IV-52, and the chemically linked conjugate SW IV-134, in a panel of four ovarian cancer cell lines. Although SW43 was effective against different ovarian cancer cell lines, SW IV-52 had minimal single-agent activity at concentrations up to 200 μM (Figure 2). Even though we noticed an augmentation of cell death with the combination of SW43 and SW IV-52, greater cytotoxicity was induced upon treatment with the chemically linked conjugate, SW IV-134 (Figure 2, p < 0.03 for all cell lines except OVCAR3, NS).

**Figure 3:**
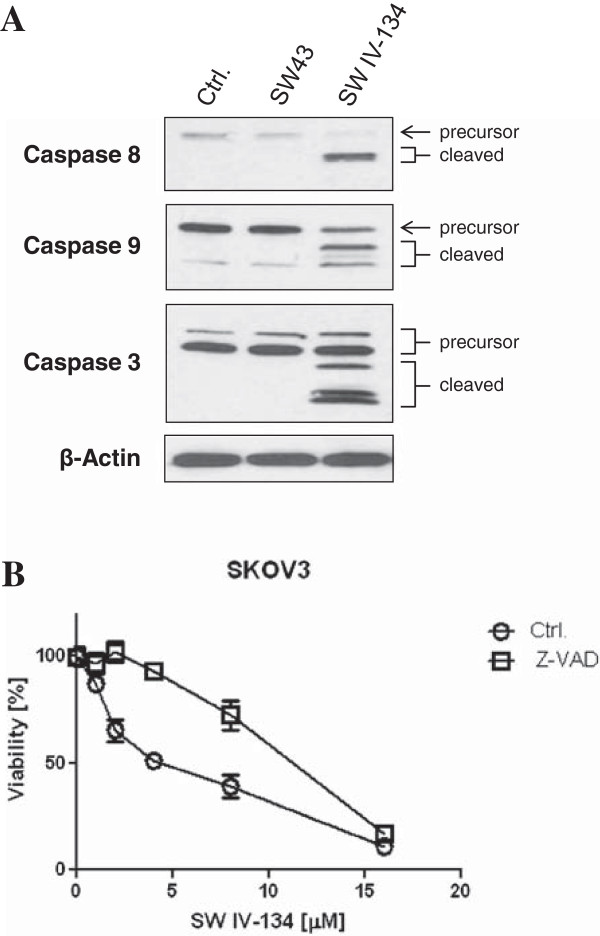
**Treatment with SW IV-134 results in caspase-mediated cell death. (A)** Cells were treated with vehicle only (Ctrl.), SW43 (10 μM), and SW IV-134 (10 μM) for 24 hours. Cell lysates were subjected to Western blot analysis, and probed with antibodies specific for the precursors and activated forms of caspases 8, 9, and 3. **(B)** SKOV3 cells were treated with SW IV-134 at indicated doses either alone or in the presence of the pan-caspase inhibitor Z-VAD-FMK (20 μM) for 18 hours. Cell viability was determined by CellTiter-Glo assays (p < 0.018, Student’s t-test).

We have previously shown that at higher concentrations, sigma-2 ligands cause cancer cell death by multiple pathways, involving caspase activation and lysosomal destabilization [13,15,22]. Herein, we demonstrate that SW IV-134 induces apoptotic cell death at substantially lower concentrations than the unconjugated sigma-2 ligand. Along these lines, activation of caspase 3, caspase 8, and caspase 9 was induced by SW IV-134, but not by SW43 in different ovarian cancer cell lines (Figure 3A, and Additional file 3: Figure S2). These caspases are well known to be the initiators and executioners of apoptotic signals generated from diverse types of stimuli [23]. Another means to investigate programmed cell death is monitoring the inversion of phosphatidylserine at the plasma membrane, which is one of the hallmarks of the early stages of apoptosis, and can be detected by cell surface binding of Annexin V [24]. Treatment with SW IV-134 in SKOV3 cells increased Annexin V binding relative to the untreated control cells in a dose dependent manner (Additional file 4: Figure S3). Additionally, SW IV-134 induced cell death was significantly reduced with the pan-caspase inhibitor Z-VAD-FMK, further pointing to the induction of apoptosis by a caspase-dependent mechanism (Figure 3B, p < 0.018).

### SW IV-134 leads to degradation of cIAP-1, cIAP-2, and activation of NF-қB

**Figure 4:**
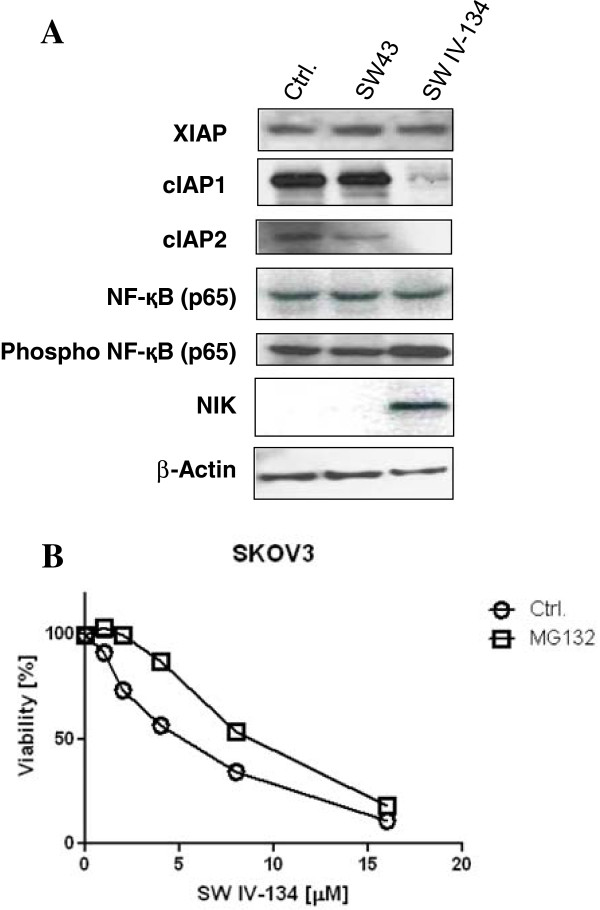
**SW IV-134 engages in SMAC-dependent pathway activation. (A)** SKOV3 cells were treated with vehicle only (Ctrl.), SW43 (10 μM), and SW IV-134 (10 μM) for 4 hours and cell lysates were subjected to Western blot analysis using antibodies against XIAP, cIAP-1, cIAP-2, NF-қB and NIK. While XIAP seems unaffected under the conditions used, cIAP-1 and cIAP-2 proteins are readily undetectable shortly post treatment. SW IV-134 treatment also leads to accumulation of NF-қB inducing kinase (NIK) and activation of NF-қB pathway. **(B)** SKOV3 cells were treated with SW IV-134 at the indicated doses either alone or in the presence of proteasome inhibitor MG132 (20 μM) for 18 hours. Cell death induction is partially rescued by MG132 and suggests a requirement for an intact proteasomal machinery to achieve efficient tumor cell killing. Cell viability was determined as described in the experimental methods (p < 0.011, Student’s t-test).

SMAC mimetics negatively regulate the caspase-inhibiting activity of IAPs [7,25]. In order to study the mechanism of SW IV-134 mediated cancer cell death, we examined its effect on IAP proteins in various ovarian cancer cell lines. Treatment with SW IV-134 led to a rapid loss of both cIAP-1 and cIAP-2 without affecting the XIAP levels (Figure 4A, and Additional file 5: Figure S4). It has been reported that cIAP-1 and cIAP-2 function as E3 ligases, responsible for the proteasomal degradation of NF-қB-inducing kinase (NIK) [26]. Under this premise, SW IV-134 mediated degradation of cIAP1 and cIAP2 would prevent the proteasomal degradation of NIK. Indeed, treatment with SW IV-134 resulted in the accumulation of NIK (Figure 4A), which was not seen with SW43 or vehicle treated cells. Additionally, treatment with SW IV-134 led to activation (phosphorylation) of NF-қB (p65) (Figure 4A). Taken together, these data suggest that SW IV-134 stimulated activation of NF-қB signaling, does not require loss of XIAP, but rather relies on the proteasomal degradation of cIAP-1 and cIAP-2. To further support this hypothesis, SW IV-134 mediated cell death was significantly reduced in the presence of the proteasome inhibitor MG132 (Figure 4B, p < 0.011).

### SW IV-134 induces TNFα-dependent cell death

**Figure 5:**
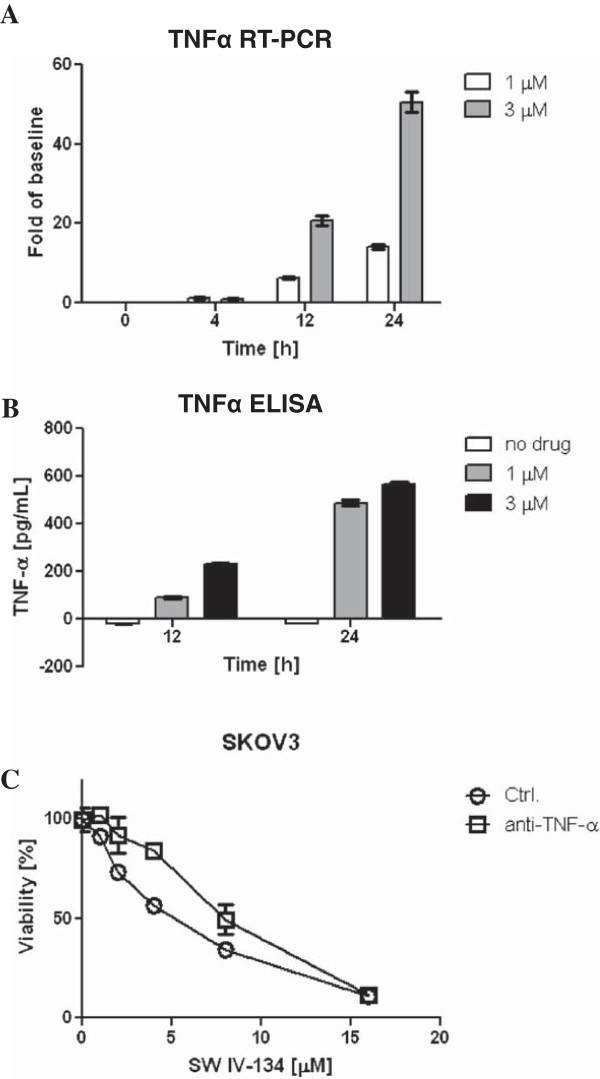
**SW IV-134 induces activation of the TNFα-dependent extrinsic death pathway. (A)** Quantitative real-time PCR analysis of TNFα mRNA expression was done on RNA samples derived from SKOV3 cells treated with SW IV-134 (1 μM and 3 μM) for indicated time periods. All values were normalized to a GAPDH internal control (p < 0.0001, one-way ANOVA). **(B)** SKOV3 cells were treated with SW IV-134 (1 μM and 3 μM) and cell-culture supernatants were collected at 4, 12, and 24 hours after treatment, and subsequently examined by anti-TNFα ELISA according to manufacturer’s instructions (p < 0.0001, one-way ANOVA). **(C)** SKOV3 cells were treated with SW IV-134 at indicated doses either alone or in the presence of anti-TNFα blocking antibody for 18 hours. Cell viability was determined as described in the experimental methods (p < 0.019, Student’s t-test).

Next, we examined the effect of SW IV-134 on the production of TNFα, a well-established target of NF-қB signaling [8]. Treatment with SW IV-134 resulted in a 20-50 fold increase in TNFα mRNA levels compared to the untreated cells (Figure 5A, p < 0.0001). The induction of TNFα mRNA was maximal at 24 hours post-treatment. We also evaluated the effect of SW IV-134 on TNFα protein secretion. SKOV3 cells were treated with SW IV-134, and conditioned medium was harvested at 4, 12, and 24 hours post treatment and analyzed by ELISA. TNFα was undetectable at 4 hours, mildly elevated at 12 hours, and highest levels were seen at the 24 hour time point, consistent with its transcriptional upregulation (Figure 5B, p < 0.0001). To investigate the functional importance of TNFα protein secretion, we treated SKOV3 cells with SW IV-134, either alone or in the presence of TNFα-blocking antibody. TNFα-blocking antibody provided significant protection from cell death induced by SW IV-134, which suggests that the pro-apoptotic activity of SW IV-134 is dependent on TNFα signaling (Figure 5C, p < 0.019).

### In vivo efficacy of SW IV-134 in an intraperitoneal xenograft model of ovarian cancer

**Figure 6:**
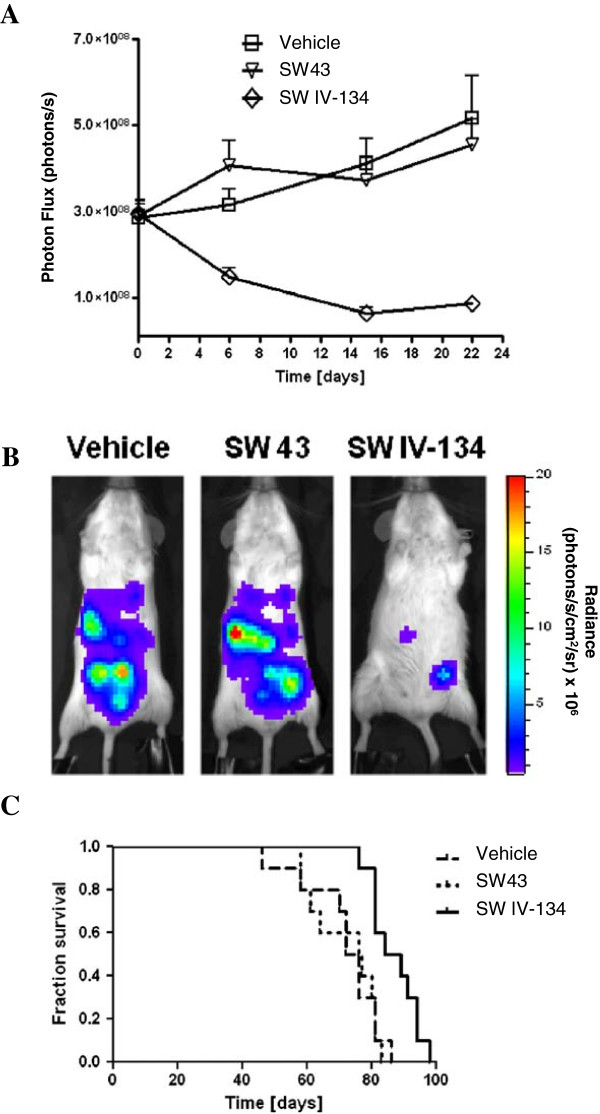
**SW IV-134 is a potent single-agent cancer drug in a peritoneal mouse xenograft model of ovarian cancer. (A)** Female SCID mice were inoculated intraperitoneally with 5 × 10^6^ SKOV3-Luc cells. Treatment was initiated with intraperitoneal injections of SW43, SW IV-134, or vehicle control 7 days after tumor cell inoculation, and continued for a total of 3 weeks. Mice were imaged once a week during the treatment period. Change in bioluminescence photon flux during treatment in each experimental arm (n = 15) was evaluated. Bioluminescent signals at each time point were averaged per treatment group (mean ± SEM). Significant differences were noted between vehicle vs. SW IV-134 and SW43 vs. SW IV-134 (p < 0.0097, one-way ANOVA). **(B)** Bioluminescent images at the end of the treatment (day 21) of one representative mouse per treatment arm (n = 15). Image intensity is displayed as radiance (photons/s/cm^2^/sr). **(C)** Kaplan-Meier survival analysis. At the end of the 3 week treatment, 5 mice from each experimental group were subjected to necropsy. The remaining 10 mice from each treatment group were followed until death to examine overall survival (p < 0.0108, Log-rank test).

The response of intraperitoneal SKOV3-Luc xenografts to treatment with SW43, SW IV-134, or vehicle-control was assessed by bioluminescence imaging. Tumor regression was evident by the end of the first week of treatment with SW IV-134 (Figure 6A, p < 0.0097). In contrast, the signal intensity increased in both the SW43 and the vehicle-only treatment groups. At the end of the 3 week treatment, the signal intensity in the SW IV-134 treatment group was significantly lower than both the SW43 and the vehicle-only treatment groups (Figure 6B). SW IV-134 treatment also conferred a survival advantage for mice in this experiment. The median survival for mice treated with SW IV-134 was 86.5 days compared to 76.5 days for mice treated with SW43 and 74 days for those treated with the vehicle only (Figure 6C, p < 0.0108).

We also assessed the potential of our drug conjugate SW IV-134 to cause adverse effects following intraperitoneal administration. The primary finding was mild to moderate peritonitis which was also seen in the SW43 and the vehicle treated mice, suggesting it to be vehicle related phenomenon. In addition, the mice treated with SW IV-134 exhibited slightly lower hemoglobin levels and higher total white blood cell count than the SW43 and the vehicle treated mice (Additional file 6: Table S2). The gross pathology and histology reports of various internal organs were not appreciably different between the SW IV-134, SW43, and the vehicle-only groups. The only exception was the presence of mild splenomegaly in SW IV-134 treated mice, which is likely due to compensatory extra-medullary hematopoiesis. Similarly, there was no significant difference between the treatment groups with regard to the comprehensive blood chemistry panel also performed at the time of necropsy (Additional file 6: Table S2). Throughout the course of the treatment, mice in all three treatment groups appeared well, and did not experience any treatment related deaths.

## Discussion

Sigma-2 receptors are preferentially expressed in proliferating tumor cells [27], and are therefore considered potential targets for a selective delivery of therapeutics into the cancer cells. Insights from the current understanding of the cell death pathways have led to the identification of several candidate proteins that could be targeted for the eradication of cancer cells. One such class of prominent cell survival factors are the inhibitors of apoptosis proteins (IAPs). IAPs are frequently overexpressed in many types of human malignancies, making them attractive targets for therapeutic intervention [28]. In the current work we have shown the feasibility of conjugating a small molecule SMAC mimetic to a sigma-2 ligand and demonstrated the effectiveness of the conjugate in treatment of ovarian cancer using in vitro and in vivo studies.

Our results indicate that SW IV-134 potentiates the sigma-2 ligand related cell death in different ovarian cancer cell lines (Figure 2). Furthermore, the cell death induced by SW IV-134 was significantly greater than that observed with a combination of SW43 and SW IV-52 (Figure 2). This is likely due to the more efficient delivery of the small molecule SMAC mimetic into the tumor cells following conjugation to the sigma-2 ligand SW43. The SMAC mimetic in turn enhances the cytotoxicity of the compound by negating the activity of two anti-apoptotic proteins, cIAP-1 and cIAP-2 (Figure 4A), thus establishing an intracellular environment that is conducive for NF-қB activation. The loss of cIAPs with SW IV-134 treatment is likely secondary to the SMAC mimetic-induced auto-ubiquitination and proteasomal degradation, and has been shown by others [29].

The NF-қB transcription factors [such as NF-қB1 (p105/p50), NF-қB2 (p100/p52), RelA(p65), ReLB, and c-ReL] are usually retained in the cytoplasm of un-stimulated cells by IқB (Inhibitor of қB) proteins [30]. In the canonical NF-қB pathway, phosphorylation of IқB by IKK2 (IқB Kinase) leads to its proteasomal degradation, allowing nuclear translocation of the transcription factors [31,32]. It has been shown that phosphorylation of NF-қB p65 is critical for the canonical NF-қB signaling [33]. On the other hand, NF-қB-inducing-kinase (NIK) is one of the key regulators of the non-canonical NF-қB pathway. NIK phosphorylates IKKα, resulting in the phosphorylation of p100, which leads to the ubiquitination and partial proteasomal degradation of p100 to its mature p52 form [34,35]. Treatment with SW IV-134 led to phosphorylation of NF-қB p65 as well as accumulation of NIK (Figure 4A), suggesting its role in activation of both canonical and non-canonical NF-қB signaling pathways. The activation of NF-қB pathways, in turn, led to the transcriptional upregulation of TNFα mRNA (Figure 5A), directly corresponding with an increase in TNFα protein (Figure 5B) and subsequent potentiation of cell death via activation of the extrinsic pathway of apoptosis, similar to what has been described by other investigators [26].

SW IV-134 reduced the tumor burden of the intraperitoneal xenografts and significantly improved survival in mice (Figure 6). A frequent criticism of many xenograft models is the location of tumor formation and its resemblance to human disease [36]. Because most ovarian cancers in humans are diagnosed at late stages, we believe that our intraperitoneal tumor model resembles more closely the advanced-stage human disease than a localized orthotopic model. In this widely metastatic intraperitoneal model, SW IV-134 demonstrated modest effectiveness, extending median survival by 10 days. Furthermore, the treatment was well tolerated with minimal adverse effects on the animals as evidenced by blood testing and histopathological analysis of various organs and tissues harvested at the end of treatment. Treatment with sigma-2 ligands has repeatedly been shown to induce apoptosis with limited off-site toxicities. This is because cancer selectivity is a key feature of our sigma-2 based platform concept, and has been demonstrated in numerous previous studies [12,15,19,21]. In one of these studies it was demonstrated that nude mice bearing either mouse 66 (mammary carcinoma) or MDA-MB-435 cells (human melanoma) injected with the fluorescently-labeled sigma-2 ligand SW120 was taken up selectively by proliferating tumor cells and not by the generally quiescent peripheral blood mononuclear cells [21]. Similar results were seen in vitro, where sigma-2 ligands were preferentially taken up by transformed cancer cells and not by the immortalized normal human HPDE cells (data not shown). Furthermore, new clinical data have recently become available that once again highlight the tendency of our drugs to localize primarily to solid tumors with negligible accumulation in the surrounding, healthy tissue [37]. These results suggest that SW IV-134 may have considerable potential for the treatment of ovarian cancers with a favorable therapeutic window.

Anti-tumor efficacy of SMAC mimetics has been reported in a variety of tumor-types by other investigators [20,38]. Furthermore, SMAC mimetics have been shown to exhibit considerable synergism in combination with a wide variety of therapeutic agents ranging from conventional therapy to TRAIL and other biologic agents [9-11,39]. Because SW IV-134 induced apoptosis targets the same mechanistic pathways as the SMAC mimetics, it can also be potentially combined with different chemotherapeutic agents to overcome resistance to therapy-induced apoptosis. We believe that the selective delivery of SMAC mimetics into the tumor cells by sigma-2 ligands (as achieved with SW IV-134) will likely enhance its on-target effects while keeping the off-target effects to a minimum. We thus envision the unique properties of SW IV-134 highly advantageous when used alone but especially in combination with existing and/or novel chemotherapeutics. As a result, we are currently in the process of exploring the tumoricidal effects of SW IV-134 in combination with different chemotherapeutic agents, to identify the most efficacious treatment regimen which could be moved forward for further clinical investigations.

## Conclusions

In summary, we have described the effectiveness and potential application of SW IV-134, a novel conjugate of sigma-2 ligand and a small molecule SMAC peptidomimetic, in the treatment of ovarian cancer. We believe this approach is particularly meritorious considering the cancer selectivity of sigma-2 ligands and the important contribution of IAPs to both de-novo and acquired treatment resistance, and therefore deserves serious consideration for future clinical development.

## Materials and methods

### Cell lines and reagents

SKOV3 cells were obtained from Dr. Robert Mach (Washington University School of Medicine, St. Louis, MO), Hey A8 and Hey A8 MDR cells from Dr. Anil Sood [40] (M.D. Anderson Cancer Center, Houston, TX), and OVCAR3 cells were purchased from American Type Culture Collection (ATCC, Manassas, VA). SKOV3 cells were labeled with a eYFP/luciferase reporter fusion protein by retroviral infection to generate SKOV3-Luc cells (G. Garg and D. Spitzer, unpublished data). Protein expression was confirmed by flow cytometry and in vitro luciferin conversion. SW43 [19] and SW IV-52 [41] were synthesized as previously reported. Synthesis of SW IV-134 is described in detail in the Additional file 7: Supplementary methods. MG-132 was purchased from Calbiochem (Billerica, MA), Z-VAD-FMK from Enzo Life Sciences (Ann Arbor, MI), and anti-TNFα antibody was purchased from R&D systems (Minneapolis, MN).

### Receptor binding assays

The sigma-1 and sigma-2 receptor binding affinities of SW IV-134 were determined as previously described [42]. Briefly, guinea pig brain (sigma-1 assay) or rat liver (sigma-2 assay) membrane homogenates (~300 μg protein) were diluted with 50 mM Tris-HCl, pH 8.0 and incubated with the radioligand {~5 nM [^3^H](+)-pentazocine (34.9 Ci/mmol; sigma-1 assay) or ~5 nM [3H](+)-DTG (58.1 Ci/mmol; sigma-2 assay)} and SW IV-134 with concentrations ranging from 0.1 nM to 10 μM in a total volume of 150 μL in 96-well plates at 25°C. After incubating for 120 minutes, the reactions were terminated by the addition of 150 μL of ice-cold wash buffer (10 mM Tris-HCl, 150 mM NaCl, pH 7.4) using a 96-channel transfer pipette (Fisher Scientific, Pittsburg, PA), and the samples harvested and filtered rapidly into a 96-well fiberglass filter plate (Millipore, Billerica, MA) that had been presoaked with 100 μL of 50 mM Tris-HCl, pH 8.0 for 1 hour. Each filter was washed three times with 200 μL of ice-cold wash buffer, and the bound radioactivity quantified using a Wallac 1450 MicroBeta liquid scintillation counter (Perkin Elmer, Boston, MA). Nonspecific binding was determined in the presence of 10 μM cold haloperidol.

### Blocking studies

SKOV3 cells (3 × 10^4^/well) were seeded into 6 cm plates for 24 hours before treatment. The cells were incubated with 5, 10, 50, and 100 μM doses of SW IV-134 for 30 minutes at 37°C. After this step, 10 nM SW120 was added to the cell culture medium containing SW IV-134. After 30 minute incubation at 37°C, cells were washed twice with phosphate buffered saline (PBS) and harvested with 0.05% trypsin EDTA (Life Technologies, Grand Island, NY). The cells were centrifuged at 1000 × g for 5 minutes and pellets washed twice with PBS. Internalization of SW120 was determined by flow cytometer (FACSCalibur, BD Biosciences, San Jose, CA).

### Annexin V binding

SKOV3 cells were treated with different concentrations of SW IV-134 (3 μM, 6 μM, and 10 μM) for 16 hours. Staining was performed using the Annexin V Apoptosis Detection Kit (BioLegend, San Diego, CA) according to the manufacturer’s instructions. The apoptosis rate was determined by flow cytometry (FACSCalibur, BD Biosciences, San Jose, CA).

### Western blot analysis

Cells were lysed in radioimmunoprecipitation assay buffer [50 mM Tris, 150 mM sodium chloride, 1.0 mM EDTA, 1% Nonidet P40, and 0.25% SDS (pH 7.0)], supplemented with complete protease inhibitor cocktail (Roche, Mannheim) and phosphatase inhibitor cocktail 1 (Sigma Chemical Co., St. Louis, MO). The protein concentration was determined using a BioRad Dc protein assay kit (Bio-Rad Laboratories, Hercules, CA). Lysates containing 30 μg of protein were run on a 12% polyacrylamide gel and transferred to a PVDF membrane (Bio-Rad Laboratories, Hercules, CA). The PVDF membrane was incubated with 5% nonfat dry milk for 1 h at room temperature, then overnight with a primary antibody at 4°C, and finally with the secondary antibody, horse-radish peroxidase-conjugated IgG, and signal visualized using the super Signal West Pico Chemiluminiscent Substrate assay kit (Pierce Biotechnology, Rockford, IL). The primary antibodies against cIAP1, cIAP2, XIAP, NIK, NF-қb (p65), phospho- NF-қB (p65), caspase3, caspase 8, and caspase 9 (all caspase-detecting antibodies react with both their precursors and cleaved [activated] forms) were purchased from Cell Signalling Technology (Danvers, MA, USA), whereas actin antibody was from Santa Cruz Biotechnology (Dallas, TX). The secondary antibodies against mouse, rabbit, and goat were also purchased from Santa Cruz Biotechnology (Dallas, TX).

### Quantitative RT-PCR

SKOV3 cells were treated with SW IV-134 (1 μM, 3 μM) or vehicle-control for 4 hours, 12 hours, and 24 hours. At the end of each treatment, cells were harvested and total RNA was isolated using TRIzol reagent (Invitrogen, Grand Island, NY) and RT-PCR performed as per the protocol described before [43]. Briefly, one microgram of RNA from each sample was reverse transcribed to cDNA with Retroscript (Ambion, Austin, TX). Resulting cDNA was diluted to an equivalent of 10 ng/μL of input RNA. Primer/probe sets for TNFα and GAPDH were purchased from Applied Biosystems (Grand Island, NY). Each reaction consisted cDNA, TaqMan Master Mix (Applied Biosystems), and primer/probe set in a total of 10 μL, following the manufacturer’s standard protocol. For each transcript/sample, triplicate reactions were done in an ABI7500FAST Sequence Detection System. TNFα data were normalized for expression with GAPDH, and results were expressed as fold change over untreated controls.

### ELISA

SKOV3 cells were treated with SW IV-134 (1 μM and 3 μM). Cell culture supernatants were collected at 4, 12, and 24 hours after treatment. Medium was collected, floating cells were removed by centrifugation at 1200 × g for 5 minutes, and samples were frozen at -20°C until analysis. TNFα ELISA was performed using a quantitative high sensitivity sandwich immunoassay from eBioscience (San Diego, CA) as per the manufacturer’s instructions.

### Analysis of cell death

Cells (1 × 10^4^) were seeded into 96 well plates. Treatment as described in the figure legends was initiated the following day. Cell viability was determined 18 hours after treatment using CellTiter-Glo Luminiscent Viability Assay (Promega, Madison, WI). Data were recorded with a SpectraMax Gemini microplate spectrofluorometer, Molecular Devices (Silicon Valley, CA).

### Caspase activation assays

Caspase 3, 8 and 9 activities were measured using Caspase-Glo® Assay Systems according to the manufacturer’s instructions (Promega, Madison, WI). Briefly, the assay systems are based on caspase-specific substrates, which are activated by cleavage, resulting in caspase specific luminescence signals. HeyA8 cells were plated at a density of 1 × 10^4^ in white 96 well, clear bottom plates for 24 hours before treatment. Cells were treated with different concentrations of SW IV-134. Caspase 3, 8 and 9 assays were performed by adding 100 μl of caspase reagents to each well. The contents were mixed using a plate shaker for 30 seconds and incubated at room temperature for 90 minutes. Luminescence signal was measured using a multi-mode microplate reader (BioTek, Winooski, VT).

### Animal studies

All studies were performed in accordance with an animal protocol approved by the Washington University Institutional Animal Care Facility. Female severe combined immunodeficient mice (SCID) were purchased from Taconic Farms (Hudson, NY) at age 6 weeks. SKOV3-Luc cells (5 × 10^6^) were inoculated intraperitoneally and mice were randomized 7 days later into one of the three treatment groups according to their baseline luciferase activity employing bioluminescence imaging (BLI). For bioluminescence imaging, mice were injected intraperitoneally with 150 μg/g D-luciferin (Biosynth, Naperville, IL) in PBS, anesthetized with 2.5% isoflurane, and imaged with a charge-coupled device (CCD) camera-based bioluminescence imaging system (IVIS 100; Caliper, Hopkinton, MA; exposure time 300 seconds, binning 16, field of view 12, f/stop 1, open filter). Signal was displayed as radiance (photons/sec/cm^2^/sr) [44].

In vivo experiments were conducted with 15 mice per treatment group. Treatment involved daily intraperitoneal injections with 90 μL of SW43 (7.5 mM stock), SW IV-134 (7.5 mM stock), or vehicle-only (25% cremophor in H_2_O) for 3 weeks. Tumor burden was monitored by bioluminescence imaging once a week during the treatment period. At the end of the treatment, 5 mice were randomly removed per treatment group and submitted for necropsy to the Washington University Department of Comparative Medicine. Blood samples were collected by intracardiac withdrawal prior to necropsy for chemistry and hematology work-up. In addition, various key organs were harvested for gross and histopathological examination. The remaining 10 mice from each treatment group were followed over time to examine overall survival. For survival studies, actual death or poor physical condition/large tumor size meeting criteria for euthanasia, constituted a death event.

### Statistical analyses

Statistical analyses and data plotting were performed using GraphPad Prism software version 5 (San Diego, CA). Results were expressed as mean ± SEM of at least 3 biological replicates. One-way ANOVA was used to analyze the differences in cell killing assays (Titer-Glow), TNFα quantification assays (RT-PCR and ELISA) and for measuring tumor sizes. The Kaplan-Meier survival curve was plotted and the difference between the groups was compared with a Log-rank test. P values < 0.05 were considered significant for all analyses.

## Abbreviations

IAP: Inhibitor of apoptosis proteins; cIAP: Cellular inhibitor of apoptosis proteins; XIAP: X-linked inhibitor of apoptosis proteins; SMAC: Second mitochondria-derived activator of caspases; NIK: NF-қB-inducing kinase.

## Competing interest

Robert Mach and William Hawkins own patent rights for the sigma-2 related drugs. All other authors declare no conflict of interest.

## Authors’ contributions

Conception and design: GG, WGH, DS. Development of methodology: GG, SV, CZ. Acquisition of data (provided animals, acquired and managed patients, provided facilities, etc.): GG, LC, CH. Analysis and interpretation of data (e.g., statistical analysis, biostatistics, computational analysis): GG, WGH, DS. Writing, review, and/or revision of the manuscript: GG, YH, DP-W, MAP, DGM, RHM, WGH, DS. Administrative, technical, or material support (i.e., reporting or organizing data, constructing databases): SV, CZ, LC, CH, YH. Study supervision: WGH, DS. All authors read and approved the final manuscript.

## Supplementary Material

Additional file 1: Table S1 Binding Affinities for Sigma-1 and Sigma-2 Receptors. The sigma-1 and sigma-2 receptor binding affinities of SW IV-134 were determined as previously described [43]. For details see also the Materials and methods section. Click here for file

Additional file 2: Figure S1 Receptor binding characteristics of the individual components of SW IV-134. SKOV3 cells were pretreated with increasing concentration of (A) SW43 and (B) SW IV-52, followed by incubation with SW120, prior to analysis by flow cytometry. SW43 prevents uptake of the fluorescently labeled sigma-2 ligand SW120 in a dose-dependent fashion, similar to the drug conjugate SW IV-134, while the SMAC mimetic SW IV-52 is nearly incapable of interfering with the uptake of SW120. Click here for file

Additional file 3: Figure S2 SW IV-134 induces caspase activation in ovarian cancer. Caspase 3, 8 and 9 activities were measured in Hey A8 cells using Caspase-Glo® Assay Systems (Promega). Hey A8 cells were treated with SW IV-134 at indicated drug concentrations for 24 hours. Caspase assays were performed by adding 100 μl lysis buffer containing the substrates for the respective caspase to be assayed. Luminescence signal intensities were recorded using a multi-mode microplate reader (BioTek). Compared to untreated controls (DMSO), cells treated with SW IV-134 responded with significant increases in caspase activities, presented as fold over DMSO control. (A) caspase 8; (B) caspase 9; and (C) caspase 3. p < 0.001 for all analyses, one-way ANOVA. Click here for file

Additional file 4: Figure S3 Flow cytometric determination of apoptosis. SKOV3 cells were treated with increasing concentrations of SW IV-134. Untreated cells served as a negative staining control. The cells were then assessed for apoptosis induction by flow cytometry following staining with propidium iodide/Annexin V. Click here for file

Additional file 5: Figure S4 SW IV-134 leads to rapid degradation of cIAP-1 in several ovarian cancer cell lines. SKOV3, Hey A8, and Hey A8 MDR cells were treated with vehicle only (Ctrl.), SW43 (10 μM), and SW IV-134 (10 μM) for 2 hours. Cell lysates were prepared and subjected to Western blot analysis using an antibody against cIAP-1, which becomes readily undetectable shortly after treatment. The same membrane was also probed for β-Actin to demonstrate equal protein loading. Click here for file

Additional file 6: Table S2 Complete blood count and serum chemistries for severe combined immunodeficient (SCID) mice treated with SW IV-134, SW43, and vehicle control. SCID mice were treated daily with intra-peritoneal injections of SW IV-134, SW43, and vehicle control for 3 weeks. At the end of treatment, blood was collected from mice in each treatment group and analyzed for hemoglobin, white blood count, and platelets. Serum chemistries were also obtained to determine the levels of liver enzymes aspartate aminotransferase (AST) and alanine aminotransferase (ALT), renally cleared metabolites blood urea nitrogen (BUN) and creatinine, as well as total protein and glucose. The mice treated with SW134 were noted to have a statistically significant decrease in hemoglobin and an increase in white blood count compared to those treated with SW43 or vehicle control. Click here for file

Additional file 7 Supplementary methods. Click here for file
