# Supplementary material for: Conjugation to a SMAC mimetic potentiates sigma-2 ligand induced tumor cell death in ovarian cancer
Source: Mol Cancer. 2014 Mar 7;13:50. doi: 10.1186/1476-4598-13-50 (PMC4015918; doi:10.1186/1476-4598-13-50)
Supplement: Additional file 1: Table S1 — Binding Affinities for Sigma-1 and Sigma-2 Receptors. The sigma-1 and sigma-2 receptor binding affinities of SW IV-134 were determined as previously described [43]. For details see also the Materials and methods section. [file 1476-4598-13-50-S1.pdf]

Supplementary Table S1

Compound	K_i (nM)[a]		Sigma-1/Sigma-2[d]
	Sigma-1[b]	Sigma-2[c]	
**SW43**	134.3 ± 11.9	7.1 ± 1.3	19
**SW IV-134**	5737 ± 476	22.6 ± 1.8	253

Table S1. Binding Affinities for Sigma-1 and Sigma-2 Receptors. The sigma-1 and sigma-2 receptor binding affinities of **SW IV-134** were determined as previously described (22). For details see also the Materials and Methods section.

[a] Mean ± S.E.M. K_i values were determined by at least three experiments.
[b] K_i for inhibiting the binding of [^{3}H](+)-pentazocine to guinea pig brain homogenates.
[c] K_i for inhibiting the binding of [^{3}H](+)-**DTG** to rat liver homogenates.
[d] K_i for sigma-1 receptors/ K_i for sigma-2 receptors.
