# Supplementary figures and images for: Conjugation to a SMAC mimetic potentiates sigma-2 ligand induced tumor cell death in ovarian cancer
Source: Mol Cancer. 2014 Mar 7;13:50. doi: 10.1186/1476-4598-13-50 (PMC4015918; doi:10.1186/1476-4598-13-50)

**A**

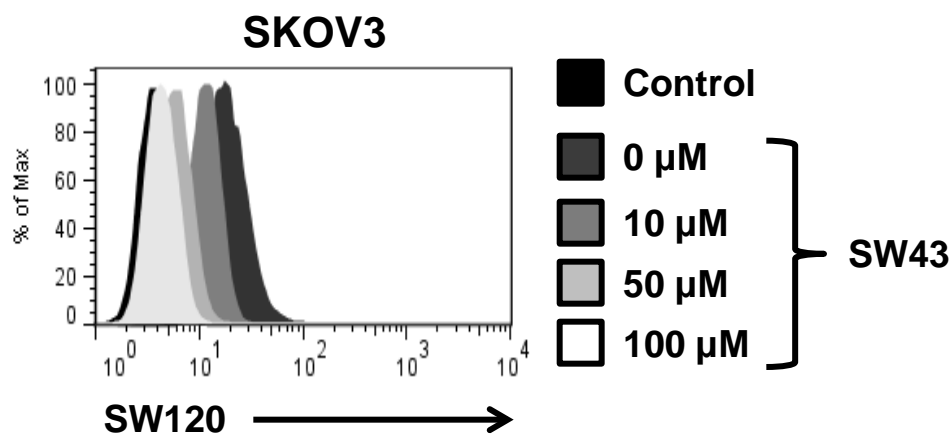

**B**

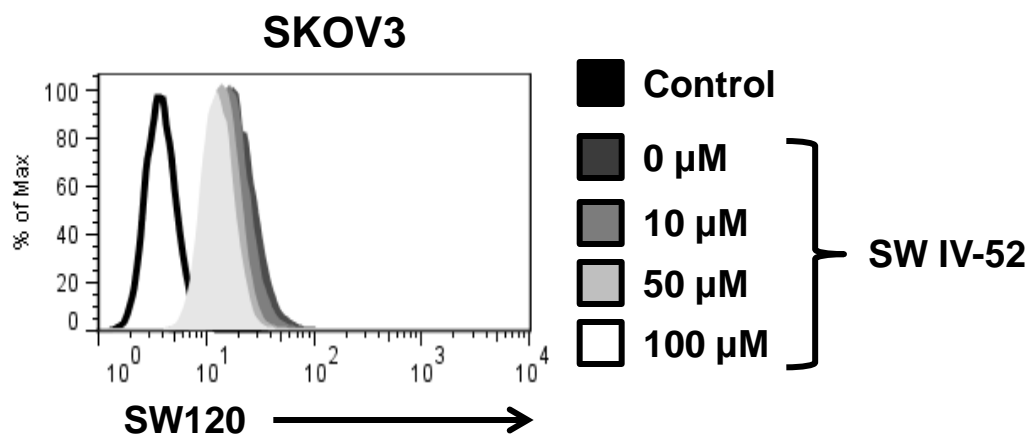

Supplement: Additional file 2: Figure S1 — Receptor binding characteristics of the individual components of SW IV-134. SKOV3 cells were pretreated with increasing concentration of (A) SW43 and (B) SW IV-52, followed by incubation with SW120, prior to analysis by flow cytometry. SW43 prevents uptake of the fluorescently labeled sigma-2 ligand SW120 in a dose-dependent fashion, similar to the drug conjugate SW IV-134, while the SMAC mimetic SW IV-52 is nearly incapable of interfering with the uptake of SW120. [file 1476-4598-13-50-S2.pdf]

**A**

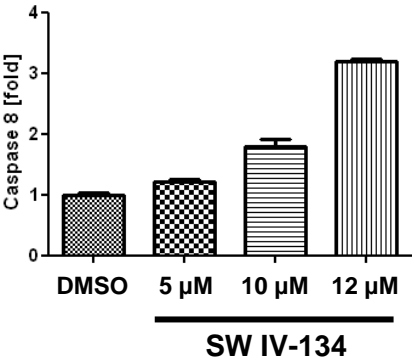

**B**

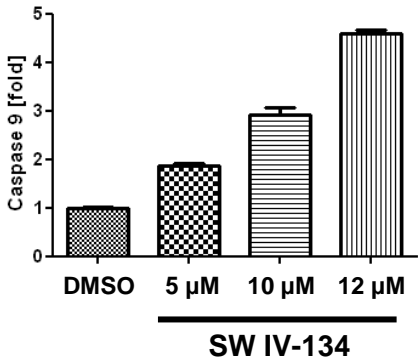

**C**

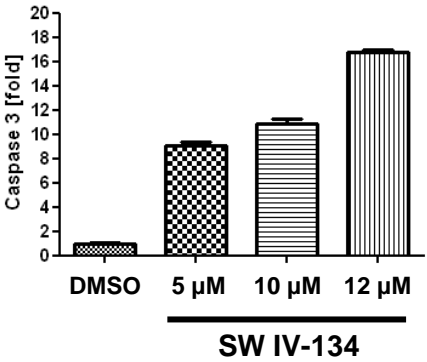

Supplement: Additional file 3: Figure S2 — SW IV-134 induces caspase activation in ovarian cancer. Caspase 3, 8 and 9 activities were measured in Hey A8 cells using Caspase-Glo® Assay Systems (Promega). Hey A8 cells were treated with SW IV-134 at indicated drug concentrations for 24 hours. Caspase assays were performed by adding 100 μl lysis buffer containing the substrates for the respective caspase to be assayed. Luminescence signal intensities were recorded using a multi-mode microplate reader (BioTek). Compared to untreated controls (DMSO), cells treated with SW IV-134 responded with significant increases in caspase activities, presented as fold over DMSO control. (A) caspase 8; (B) caspase 9; and (C) caspase 3. p < 0.001 for all analyses, one-way ANOVA. [file 1476-4598-13-50-S3.pdf]

Supplementary Figure S3

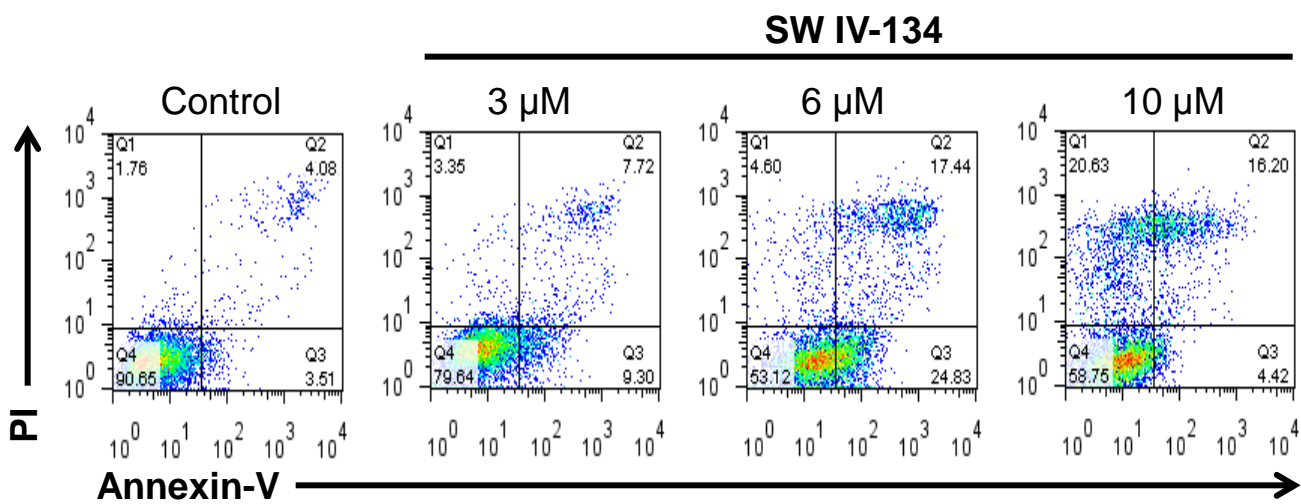

Supplement: Additional file 4: Figure S3 — Flow cytometric determination of apoptosis. SKOV3 cells were treated with increasing concentrations of SW IV-134. Untreated cells served as a negative staining control. The cells were then assessed for apoptosis induction by flow cytometry following staining with propidium iodide/Annexin V. [file 1476-4598-13-50-S4.pdf]

Supplementary Figure S4

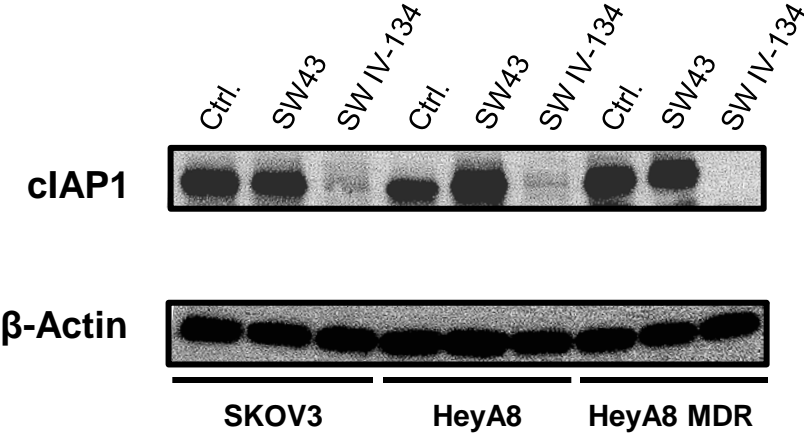

Supplement: Additional file 5: Figure S4 — SW IV-134 leads to rapid degradation of cIAP-1 in several ovarian cancer cell lines. SKOV3, Hey A8, and Hey A8 MDR cells were treated with vehicle only (Ctrl.), SW43 (10 μM), and SW IV-134 (10 μM) for 2 hours. Cell lysates were prepared and subjected to Western blot analysis using an antibody against cIAP-1, which becomes readily undetectable shortly after treatment. The same membrane was also probed for β-Actin to demonstrate equal protein loading. [file 1476-4598-13-50-S5.pdf]
