# Supplementary material for: Conjugation to a SMAC mimetic potentiates sigma-2 ligand induced tumor cell death in ovarian cancer
Source: Mol Cancer. 2014 Mar 7;13:50. doi: 10.1186/1476-4598-13-50 (PMC4015918; doi:10.1186/1476-4598-13-50)
Supplement: Additional file 6: Table S2 — Complete blood count and serum chemistries for severe combined immunodeficient (SCID) mice treated with SW IV-134, SW43, and vehicle control. SCID mice were treated daily with intra-peritoneal injections of SW IV-134, SW43, and vehicle control for 3 weeks. At the end of treatment, blood was collected from mice in each treatment group and analyzed for hemoglobin, white blood count, and platelets. Serum chemistries were also obtained to determine the levels of liver enzymes aspartate aminotransferase (AST) and alanine aminotransferase (ALT), renally cleared metabolites blood urea nitrogen (BUN) and creatinine, as well as total protein and glucose. The mice treated with SW134 were noted to have a statistically significant decrease in hemoglobin and an increase in white blood count compared to those treated with SW43 or vehicle control. [file 1476-4598-13-50-S6.pdf]

Supplementary Table S2

	SW IV-134		SW43		Vehicle		p-value
	Mean	SEM	Mean	SEM	Mean	SEM	
Hemoglobin (g/dl)	9.5	0.3	11.7	0.5	12.8	0.2	0.02
WBC (10^3 /µl)	19.3	3.7	3.2	1.0	3.4	0.5	0.02
Platelet (10^3 /µl)	1063.8	119.2	999.3	142.0	861.7	17.4	0.7
BUN (mg/dl)	24.8	1.1	22.3	2.3	27.0	1.5	0.4
Creatinine (mg/dl)	0.4	0.03	0.3	0.02	0.4	0.03	0.6
ALT (u/L)	84.0	15.5	56.0	7.6	70.7	12.9	0.4
AST (u/L)	91.4	23.4	104.7	50.3	72.7	17.6	1.0
Total bilirubin (mg/dl)	0.1	0	0.1	0.03	0.1	0	0.3
Glucose (mg/dl)	103.6	10.4	170.3	28.9	168.3	16.3	0.06
Total protein (g/dl)	6.0	0.2	5.8	0.2	5.9	0.07	0.8

Table S2: Complete blood count and serum chemistries for SCID mice treated with SW IV-134, SW43, and vehicle. SCID mice were treated daily with intra-peritoneal injections of SW134, SW43, and vehicle control for 3 weeks. At the end of treatment, blood was collected from mice in each treatment group and analyzed for hemoglobin, white blood count, and platelets. Serum chemistries were also obtained to determine the levels of liver enzymes aspartate aminotransferase (AST) and alanine aminotransferase (ALT), renally cleared metabolites blood urea nitrogen (BUN) and creatinine, as well as total protein and glucose. The mice treated with SW IV-134 were noted to have a statistically significant decrease in hemoglobin and an increase in white blood count compared to those treated with SW43 or vehicle control.
