# Supplementary material for: Conjugation to a SMAC mimetic potentiates sigma-2 ligand induced tumor cell death in ovarian cancer
Source: Mol Cancer. 2014 Mar 7;13:50. doi: 10.1186/1476-4598-13-50 (PMC4015918; doi:10.1186/1476-4598-13-50)
Supplement: Additional file 7 — Supplementary methods. [file 1476-4598-13-50-S7.pdf]

Supplementary Methods

Chemistry

The synthesis of the target compound **SW IV-134** was outlined in scheme 1. Reaction of **1**[1] and Boc-L-Ala-OH in the presence of EDCI, DIPEA and HOBt, followed by removal of the Boc protecting group gave compound **2**. Compound **3**[2] was treated with 10-bromo-1-decanol to give compound **4**. Oxidation of alcohol **4** with Dess-Martin periodinane gave the aldehyde **5**. Finally, reductive amination of compounds **2** and **5** with sodium borohydride gave the desired product **SW IV-134** in 22 % yield (overall).

Experimental Section

^{1}H NMR spectra were recorded on a Varian 300 MHz NMR spectrometer. Chemical shifts are reported in δ values (parts per million, ppm) relative to an internal standard of tetramethylsilane (TMS). The following abbreviations are used for multiplicity of NMR signals: br s = broad singlet, d = doublet, dd = doublet of doublets, m = multiplet, q = quartet, s = singlet, t = triplet. Melting points were determined on an Electrothermal melting point apparatus and are uncorrected. Elemental analyses were performed by Atlantic Microlab, Inc., Norcross, GA and were within ± 0.4% of the calculated values. All reactions were carried out under an inert atmosphere of nitrogen. Abbreviation: Alanine (Ala), t-butoxycarbonyl (Boc), *N,N*-diisopropylethylamine (DIPEA), 1-ethyl-3-(3-dimethylaminopropyl)carbodiimide (EDCI), 1-hydroxybenzotriazole (HOBt).

(*S*)-1-((*S*)-2-((*S*)-2-Aminopropanamido)-3,3-dimethylbutanoyl)-*N*-((*R*)-1,2,3,4-tetrahydronaphthalen-1-yl)pyrrolidine-2-carboxamide (2). The reaction of **1** and Boc-L-Ala-OH (follow the procedure in Reference 1) gave **2** as a light brown semi-solid (83% yield). ^{1}H

NMR ($CDCl_3$) δ 7.93 (d, *J* = 9.4 Hz, 1H), 7.22-7.27 (m, 2H), 7.04-7.14 (m, 3H), 5.11-5.14 (m, 1H), 4.57 (dd, *J* = 8.2 and 2.7 Hz, 1H), 4.53 (d, *J* = 9.7 Hz, 1H), 3.78-3.85 (m, 1H), 3.61-3.68 (m, 1H), 3.45 (q, *J* = 7.1 Hz, 1H), 2.73-2.79 (m, 2H), 2.44-2.49 (m, 1H), 2.12-2.18 (m, 1H), 1.95-2.01 (m, 2H), 1.81-1.90 (m, 4H), 1.56 (br s, 2H), 1.32 (d, *J* = 7.1 Hz, 3H), 0.85 (s, 9H).

9-(10-Hydroxydecyl)-9-azabicyclo[3.3.1]nonan-3-yl (2-methoxy-5-methylphenyl) carbamate (4). A mixture of amine **3**, 10-bromo-1-decanol (1.3 equiv), potassium iodide (1.3 equiv), and potassium carbonate (5 equiv) in acetonitrile was stirred at reflux overnight. After filtration, volatile components were evaporated in vacuo. The resulting residue was purified by column chromatography to give **4** as light yellow liquid (72% yield). ^{1}H NMR ($CDCl_3$) δ 7.95 (s, 1H), 7.13 (s, 1H), 6.73-6.79 (m, 2H), 5.10-5.16 (m, 1H), 3.84 (s, 3H), 3.64 (t, *J* = 6.7 Hz, 2H), 3.07-3.11 (m, 2H), 2.45-2.61 (m, 4H), 2.29 (s, 3H), 2.12-2.22 (m, 1H), 1.90-1.96 (m, 2H), 1.23-1.59 (m, 20H).

9-(10-Oxodecyl)-9-azabicyclo[3.3.1]nonan-3-yl(2-methoxy-5-methylphenyl) carbamate (5). A solution of **4** in dichloromethane was added to a stirred solution of Dess-martin periodinane (1.2 equiv) in dichloromethane. After stirring at room temperature overnight, the reaction mixture was diluted with ether, and poured into a saturated solution of sodium bicarbonate. After the mixture was stirred for 15 min, the ether layer was separated, dried over sodium sulfate and evaporated to give **5** as light yellow liquid (75% yield). ^{1}H NMR ($CDCl_3$) δ 9.76 (s, 1H), 7.94 (s, 1H), 7.13 (s, 1H), 6.73-6.79 (m, 2H), 5.10-5.16 (m, 1H), 3.84 (s, 3H), 3.18-3.22 (m, 2H), 2.40-2.70 (m, 5H), 2.29 (s, 3H), 1.94-2.24 (m, 3H), 1.27-1.61 (m, 20H).

(*1R,3S,5S*)-9-(10-(((*S*)-1-(((*S*)-3,3-dimethyl-1-oxo-1-((*S*)-2-(((*R*)-1,2,3,4-tetrahydronaphthalen-1-yl)carbamoyl)pyrrolidin-1-yl)butan-2-yl)amino)-1-oxopropan-2-yl)amino)decyl)-9-azabicyclo[3.3.1]nonan-3-yl(2-methoxy-5-methylphenyl)carbamate (SW IV-134). A solution of amine **2** (1 equiv) in dichloromethane was added to a solution of aldehyde **5** in dichloromethane. After stirring for 5 h, the solvent was evaporated. The resulting residue was dissolved in ethanol, and sodium borohydride (2.5 equiv) was added. After stirring for 4 h, the reaction was quenched with 10% aqueous hydrochloric acid and evaporated. The resulting residue was basified with 10% aqueous sodium hydroxide, and extracted with dichloromethane. Purification by column chromatography gave **SW IV-134** as an off-white powder (22% yield, overall), mp 73-74 ^{0}C. ^{1}H NMR ($CDCl_3$) δ 7.80-7.95 (m, 2H), 7.23-7.27 (m, 2H), 7.03-7.14 (m, 4H), 6.73-6.79 (m, 2H), 5.11-5.17 (m, 2H), 4.49-4.60 (m, 2H), 3.84 (s, 3H), 3.60-3.64 (m, 1H), 3.05-3.13 (m, 3H), 2.73-2.78 (m, 2H), 2.40-2.58 (m, 6H), 2.29 (s, 3H), 1.83-2.17 (m, 11H), 1.20-1.52 (m, 26H), 0.84 (s, 9H). Anal. ($C_{51}H_{78}N_6O_6 \cdot H_2O$) calcd C, 68.89; H, 9.07; N, 9.45. Found C, 69.12; H, 8.87; N, 9.42.

References

1. Yang C, Davis JL, Zeng R, Vora P, Su X, Collins LI, et al. Antagonism of inhibitor of apoptosis proteins increases bone metastasis via unexpected osteoclast activation. Cancer Discov. **2013**;3:212-23.
2. Mach, R.H.; Wheeler, K.T.; Blair, S.; Yang, B.; Day, C.S.; Blair, J.B.; Choi, S.; Kung, H.K. Preparation of a technetium-99m SPECT agent for imaging the sigma-2 receptor status of solid tumors. J. Labelled Cpd. Radiopharm. **2001**, *44*, 899-908.

Scheme 1.

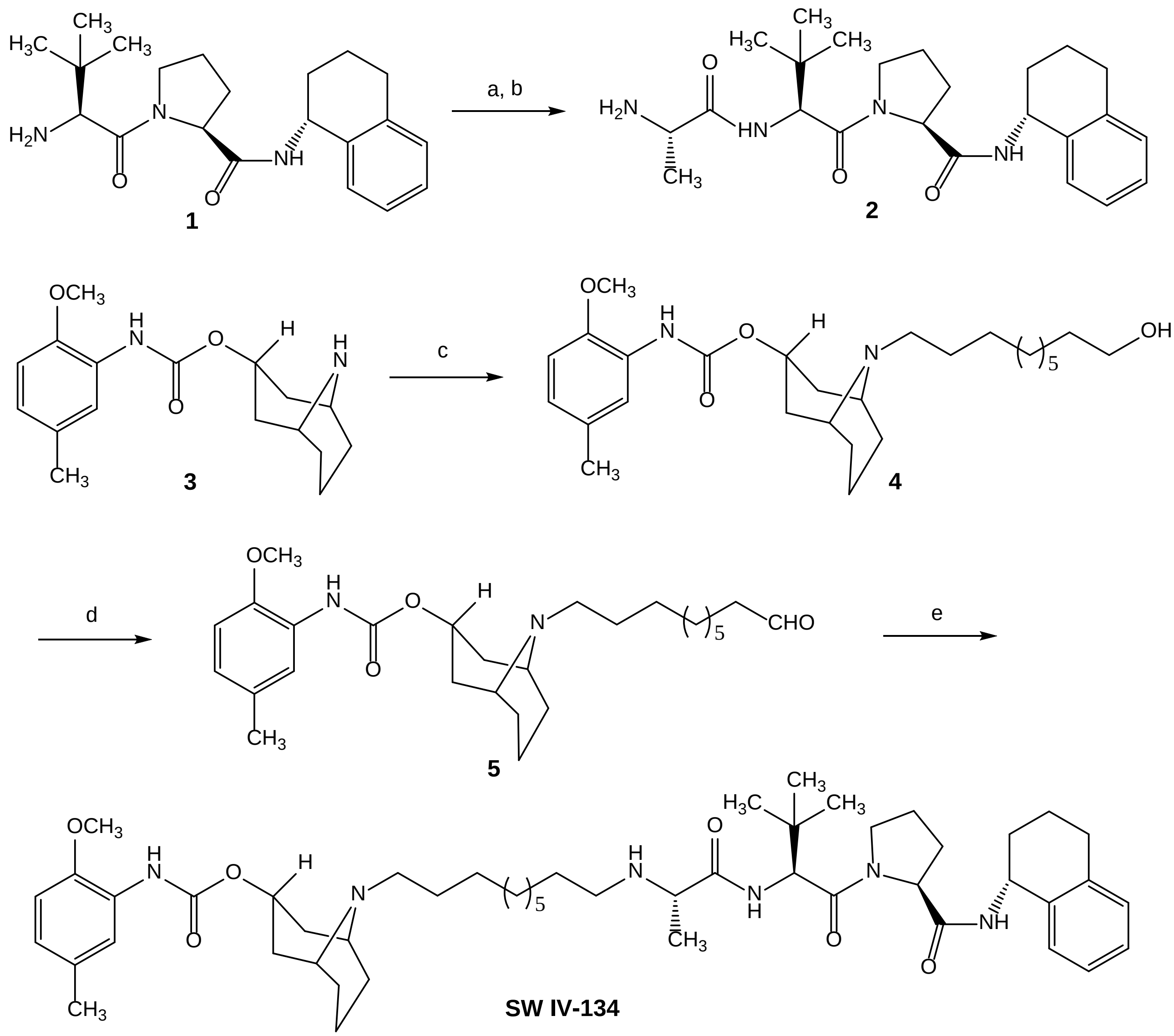

Reagents: (a) Boc-L-Ala-OH, EDCI, DIPEA, HOBt, DMF; (b) TFA, CH_2Cl_2; (c) 10-bromo-1-decanol, KI, K_2CO_3, CH_3CN, heat; (d) Dess-Martin periodinane, CH_2Cl_2; (e) 1). **2**, CH_2Cl_2; 2). $NaBH_4$, EtOH
